# Physicochemical Characterization, Antioxidant and Anticancer Activity Evaluation of an Acidic Polysaccharide from *Alpinia officinarum* Hance

**DOI:** 10.3390/molecules29081810

**Published:** 2024-04-16

**Authors:** Huan Wen, Yangjun Kuang, Xiuxia Lian, Hailong Li, Mingyan Zhou, Yinfeng Tan, Xuguang Zhang, Yipeng Pan, Junqing Zhang, Jian Xu

**Affiliations:** 1 Hainan Provincial Key Laboratory for Research and Development of Tropical Herbs, Haikou Key Laboratory of Li Nationality Medicine, School of Pharmacy, Hainan Medical University, Haikou 571199, China; 17330924995@163.com (H.W.); 17803680531@163.com (Y.K.); 18889528846@163.com (X.L.); lihailong@hainmc.edu.cn (H.L.); hy020705@hainmc.edu.cn (Y.T.); 15041258515@163.com (X.Z.); 2 Hepatobiliary and Liver Transplantation Department of Hainan Digestive Disease Center, The Second Affiliated Hospital of Hainan Medical University, Haikou 570311, China; zhoumingyan21403@163.com

**Keywords:** *Alpinia officinarum* Hance, acidic polysaccharide, plant polysaccharide, physicochemical characterization

## Abstract

AHP-3a, a triple-helix acidic polysaccharide isolated from *Alpinia officinarum* Hance, was evaluated for its anticancer and antioxidant activities. The physicochemical properties and structure of AHP-3a were investigated through gel permeation chromatography, scanning electron microscopy (SEM), Fourier transform infrared spectroscopy, and nuclear magnetic resonance (NMR) spectroscopy. The weight-average molecular weight of AHP-3a was 484 kDa, with the molar percentages of GalA, Gal, Ara, Xyl, Rha, Glc, GlcA, and Fuc being 35.4%, 21.4%, 16.9%, 11.8%, 8.9%, 3.1%, 2.0%, and 0.5%, respectively. Based on the results of the monosaccharide composition analysis, methylation analysis, and NMR spectroscopy, the main chain of AHP-3a was presumed to consist of (1→4)-α-D-GalpA and (1→2)-α-L-Rhap residues, which is a pectic polysaccharide with homogalacturonan (HG) and rhamnogalacturonan-I (RG-I) structural domains containing side chains. In addition, the results of the antioxidant activity assay revealed that the ability of AHP-3a to scavenge DPPH, ABTS, and OH free radicals increased with an increase in its concentration. Moreover, according to the results from the EdU, wound healing, and Transwell assays, AHP-3a can control the proliferation, migration, and invasion of HepG2 and Huh7 hepatocellular carcinoma cells without causing any damage to healthy cells. Thus, AHP-3a may be a natural antioxidant and anticancer component.

## 1. Introduction

Oxidative stress is a pathological state that represents a state of imbalance of oxidative and antioxidant actions in the body, in a situation where numerous free radicals accumulate [1,2]. These accumulated free radicals can damage organelles and biomolecules within the cells [3] and, thereby, directly or indirectly induce various acute and chronic diseases such as cancer, cardiovascular diseases, and cataracts [4]. Cancer is a major threat to human health and is one of the major causes of high mortality worldwide [5]. Currently, cancer treatment mainly includes surgery, radiation therapy, chemotherapy, and molecular targeted therapy [6]; however, chemotherapeutic drugs are toxic and cause damage to the body’s organs [7]. In addition, synthetic antioxidants have poor thermal stability and highly toxic side effects [8]. Therefore, safe natural products with low toxic side effects must be developed to harness their free radical-scavenging and anticancer abilities.

Polysaccharides are important macromolecules in living beings and are among the fundamental chemicals required for the proper functioning of life [9,10]. They have been reported to exhibit biological effects, such as antioxidant, anticancer, immunological regulation, and antivirus activities [11,12,13,14]. Considering the wide application of polysaccharides across food, pharmaceuticals, healthcare, and other industries, increasing numbers of studies are being conducted on them [9]. Numerous substances contain polysaccharides, which form when aldose or ketose is polymerized in a certain ratio by glycosidic bonds [15]. Different polysaccharide components from the same plant source have varying monosaccharide compositions, molecular weights, and biological activities [16]. Consequently, studies on various polysaccharide components from the same herb have enhanced our understanding of their structure and biological activities, as well as encouraging their further development and application in contemporary medicine.

*Alpinia officinarum* Hance, a plant belonging to the *Zingiberaceae* family, possesses biological functions, including antioxidant, anticancer, and gastric mucosa protective activities. It is a significant member of the medicinal and edible herbaceous family [17]. *A. officinarum* is abundant in active components, such as flavonoids, diarylheptanoids, and polysaccharides, with a huge number of polysaccharides (20.25 ± 1.11%) [18]. Wang et al. noted that the *A. officinarum* crude polysaccharide exhibited strong antioxidant activity [19]. On investigating the biological activity of the *A. officinarum* crude polysaccharide, Hailiwu et al. found that the polysaccharide could successfully suppress the growth of the murine forestomach carcinoma cell line (MFC) stomach cancer xenograft in mice [20]. Following a comprehensive structural examination, Ni et al. reported that the neutral polysaccharide of *A. officinarum* possesses immunomodulatory properties [21]. Most of these past reports are focused on *A. officinarum* crude and neutral polysaccharides, and there have been no studies on the structural properties and biological activities of *A. officinarum* acidic polysaccharides.

Consequently, in this study, acidic polysaccharide (AHP-3a) was isolated and purified mainly from the rhizome of *A. officinarum* Hance (galangal), and the purified and refined structure was characterized with the help of Fourier transform infrared spectroscopy (FT-IR), methylation, monosaccharide composition, and nuclear magnetic resonance (NMR) spectroscopy, while the antioxidant effect of AHP-3a was assessed to determine the structure and antioxidant activity of polysaccharides isolated from galangal. The anticancer activity of AHP-3a was evaluated through cexperiments. The results obtained provide new information on the structure and bioactivity of galangal acidic polysaccharides. The present results provide a reliable reference for the further development and utilization of galangal polysaccharides.

## 2. Results

### 2.1. Isolation and Purification of AHP-3a

**Figure 1:**
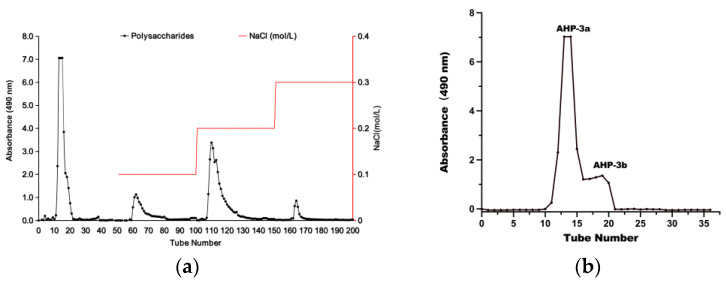
Isolation and purification of polysaccharides; (**a**) Elution curve of AHP on DEAE-52 cellulose column column; (**b**) Elution profile of AHP-3a from a Sephadex G-100 gel-filtration column.

DEAE-52 cellulose is a widely used anion exchange chromatography material, because of the presence of charged groups on its surface that can efficiently exchange with the mobile phase and sample to achieve effective chromatographic separation [22]. This feature is highly beneficial in polysaccharide purification and separation, especially for separating neutral and acidic polysaccharides. Figure 1a presents the successful fractionation of the crude polysaccharide into four portions, namely AHP-1, AHP-2, AHP-3, and AHP-4, which were obtained through elution with different NaCl concentrations. AHP-3 was further purified using a Sephadex G-100 column, as depicted in Figure 1b. This step yielded two fractions, namely AHP-3a, and AHP-3b. Notably, AHP-3a exhibited a higher peak area than AHP-3b, indicating that AHP-3a was the major fraction of AHP-3. Accordingly, AHP-3a was collected and freeze dried for further analysis.

### 2.2. UV–Vis Spectroscopy

**Figure 2:**
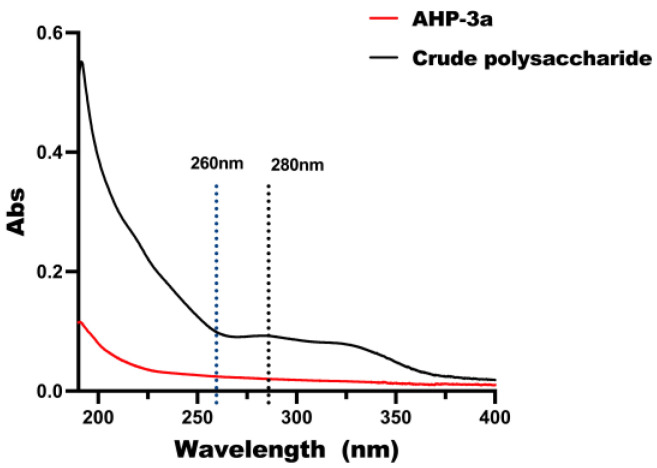
The UV spectrum of AHP-3a before and after purification.

The full-band UV scan of the polysaccharide before purification is depicted in Figure 2. These scans exhibited absorption peaks within the 260–280 nm range and represent the nucleic acid and proteins present within the crude polysaccharide sample. In contrast, AHP-3a showed no sensible absorption signals at the wavelengths of 260 nm and 280 nm in the UV scanning curves. This observation confirms that nucleic acid and protein were completely removed from the purified AHP-3a sample, thereby validating the effectiveness of the purification process.

### 2.3. Chemical and Monosaccharide Compositions of AHP-3a

**Figure 3:**
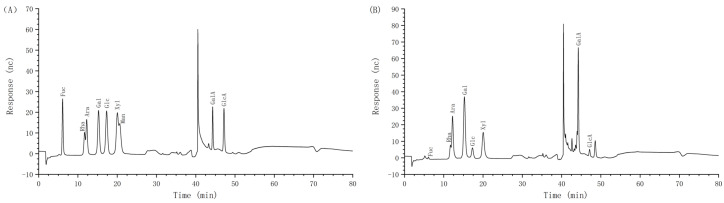
Monosaccharide analysis of AHP-3a. Monosaccharide standards (**A**); monosaccharide composition of AHP-3a (**B**).

The chemical composition of AHP-3a was determined through comprehensive analysis (Table 1). According to the results, AHP-3a contains neutral polysaccharides (28.00%), glyoxylates (45.96%), and total phenols (8.16%). Notably, AHP-3a was confirmed to be devoid of any protein content, which was consistent with our observation from the UV full wavelength scans. Table 1 and Table 2, and Figure 3, present the results of the monosaccharide composition analysis. AHP-3a was mainly composed of Rha, Ara, Gal, Xyl, and GalA, having molar percentages of 8.9%, 16.9%, 21.4%, 11.8%, and 35.4%, respectively, with small amounts of Fuc (0.5%) and GlcA (2.0%), and no Man. These findings suggest that AHP-3a is an acidic polysaccharide, with high GalA levels. Notably, our findings differ significantly from those of previous studies on the galangal neutral polysaccharide, which are composed solely of Glu and do not contain glyoxylates and total phenols [19]. These discrepancies may be attributed to the differences in the origin and selection of the components. Therefore, further studies on AHP-3a are warranted to elucidate its structural properties.

### 2.4. Molecular Weight Analysis of AHP-3a

**Figure 4:**
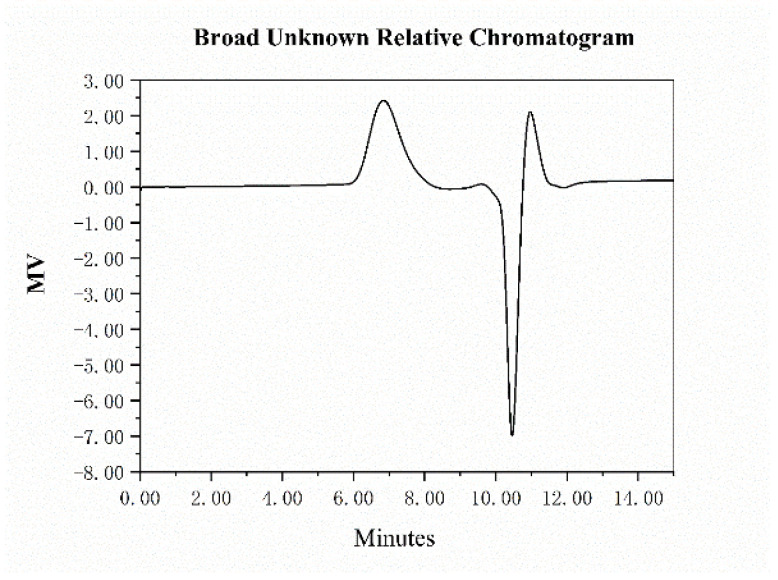
Molecular weight analysis of AHP-3a.

The GPC technique is widely used for determining the molecular weight and molecular weight distribution of polysaccharides [23]. A high-molecular-weight acidic polysaccharide fraction was recorded in this study, which was denoted as AHP-3a. As shown in Figure 4 and Table 3. It has a molecular weight of 5.83 × 10^5^ Da and a narrow molecular weight distribution, as confirmed by the calculation of the Mn and weight-average molecular weight values of 2.73 × 10^5^ and 4.84 × 10^5^ Da, respectively. This finding indicated the successful extraction of high-molecular-weight polysaccharides from galangal. Furthermore, the polydispersity index of AHP-2 was 1.77, which suggested a homogeneous particle distribution for AHP-3a.

### 2.5. SEM Analysis of AHP-3a

**Figure 5:**
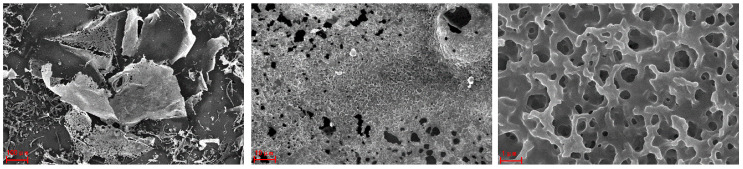
SEM micrographs of AHP-3a at the magnifications of 100×, 1000×, and 10,000×.

The SEM technique is potent and especially useful for analyzing the surface morphology and ultrastructure of polysaccharides [24]. Figure 5 presents the SEM images of AHP-3a at 100×, 1000×, and 10,000× magnifications. At 100× magnification, AHP-3a appears to have an irregular lamellar structure, surrounded by a few strips and granular pieces, and, at 1000× and 10,000× magnification, the AHP-3a surface contains numerous honeycomb-like holes. Notably, these holes may provide the polysaccharides with an increased specific surface area, more free radical active sites, and/or the inhibition of the interaction between free radicals [25], thereby possibly improving the in vitro free radical-scavenging ability of the polysaccharides.

### 2.6. Congo Red Analysis of AHP-3a

**Figure 6:**
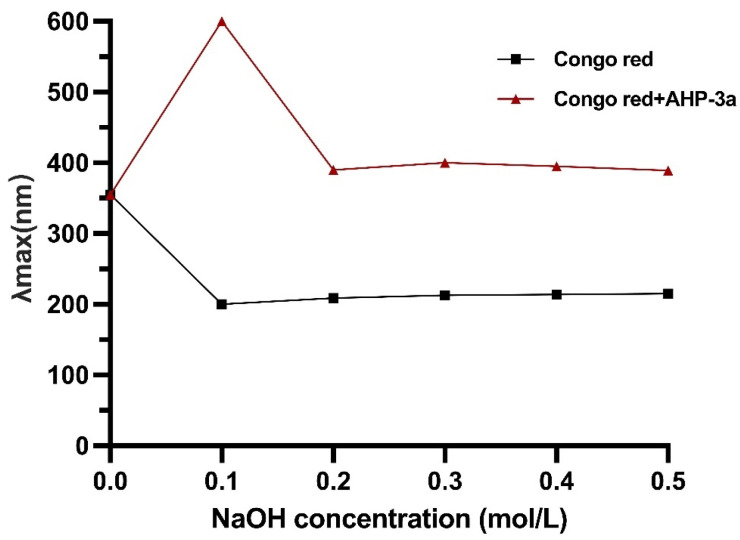
Congo red analysis of AHP-3a.

The Congo red test is a reliable method for detecting the triple-helix structure of polysaccharides, which exhibit superior biological activity, including anticancer potential [26]. Our results revealed that AHP-3a has a triple-helix conformation (Figure 6), as evidenced by the maximum absorption wavelength of 600 nm of AHP-3a at 0.1 mol/L NaOH and the redshift in the absorption wavelength range of 355–600 nm when compared with Congo red. Thus, AHP-3a is a polysaccharide with a triple-helix structure. This structure possibly confers anticancer properties on AHP-3a, which warrants further investigation.

### 2.7. FT-IR Spectra Analysis of AHP-3a

**Figure 7:**
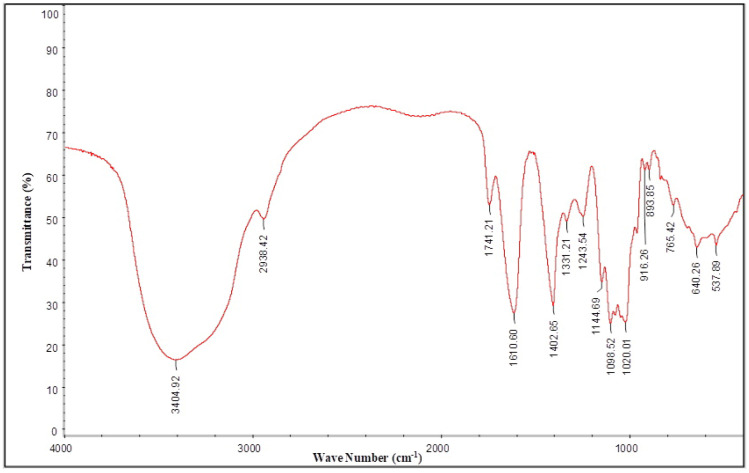
FT-IR spectroscopy of AHP-3a.

The strong absorption peak at 3404 cm^−1^ was attributable to the O–H stretching vibration of the sugar ring (Figure 7). The absorption peak at 2938 cm^−1^ was attributable to the C–H stretching vibration. The absorption peak at 1741 cm^−1^ was a clear absorption peak of the C=O stretching vibration, which indicated that a large amount of glyoxylate was present in the polysaccharide sample [27]. This finding was consistent with the experimental results of the glyoxylate content determination. The absorption peaks at 1610 and 1402 cm^−1^ were attributable to a C–O stretching vibration and a C–H or O–H bending vibration, respectively [28,29]. The absorption peaks at 1331 and 1243 cm^−1^ were attributable to the C–H bending vibration and an elastic vibration of the acetyl group, respectively [30]. The absorption peaks in the 1200–1000 cm^−1^ range were attributable to the C–O–C and C–O–H stretching vibrations [31]. The absorption peaks in the 1000–800 cm^−1^ range were characteristic of furanose residues [32]. The absorption peak at 893 cm^−1^ indicated that the polysaccharide was a furanose, with a β-configuration glycosidic bond [33]. The absorption peaks at 640 and 537 cm^−1^ were attributable to CCO deformation vibrations [34]. The results from the infrared spectra indicated that the sample mainly consisted of pyranose rings, which are acidic polysaccharides with a β-configuration glycoside.

### 2.8. Methylation Analysis of AHP-3a

Methylation analysis is currently the most effective method of determining how polysaccharide glycosidic bonds are linked [35]. Table 4 presents the linkage patterns of AHP-3a glycosidic bonds, which were determined through methylation analysis. AHP-3a has a total of 17 glycosidic bonds, namely T-Araf, T-Rhap, 1,3-Araf, 1,2-Rhap, 1,4-Xylp, T-GlcpA, T-Galp, T-GalpA, 1,2,4-Rhap, 1,4-GalpA, 1,4-Galp, 1,4-Glcp, 1,3-Galp, 1,6-Galp, 1,3,4-GalpA, 1,2,4-GlcpA, and 1,3,6-Galp, with the 1,4-GalpA unit being a major component (32.634%). Based on these sugar residues, AHP-3a is assumed to contain HG-type and RG-I-type pectin structural domains. The structures of these domains warrant further analyses in conjunction with the NMR results.

**Figure 8:**
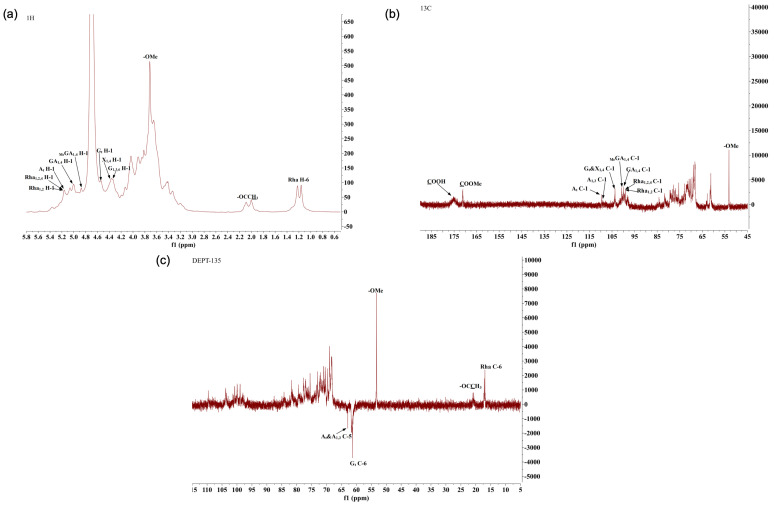
1H NMR (**a**), 13C NMR (**b**), and DEPT−135 (**c**) spectra of AHP−3a.

**Figure 9:**
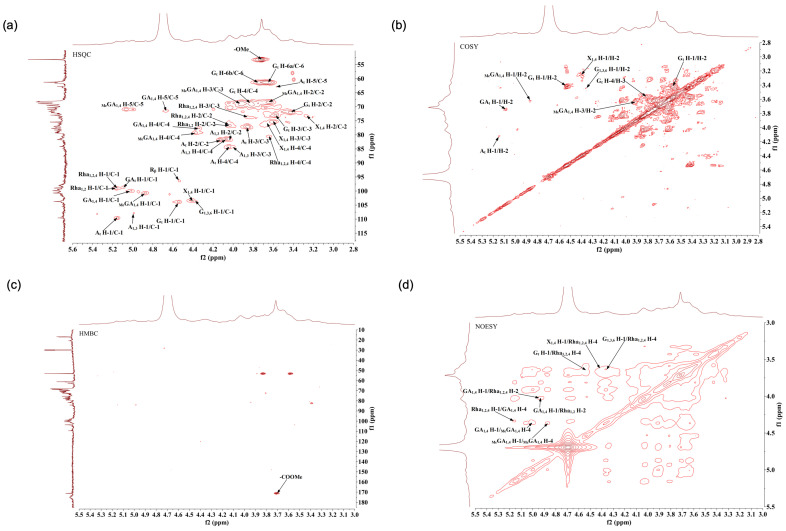
HSQC (**a**), COSY (**b**), HMBC (**c**), and NOESY (**d**) spectra of AHP-3a.

To further explore the structural features of AHP-3a, 1D and 2D NMR measurements were conducted, and their spectra are presented in Figure 8 and Figure 9, respectively.

In Figure 8c, a clear inverted peak signal can be observed in the δ 60–70 region, which indicates that the sugar residue contains a -CH_2_- group [36].

Some characteristic signal peaks were detected (Figure 8a,b). The resonance signal observed in the δ 1.10–1.30 region in the high field was attributable to the characteristic chemical shift of the methyl proton H-6 in the Rha residue. Furthermore, a unique deoxygenated structure of the methyl carbon C-6 of rhamnose was observed at δ 16.99 [37], as evidenced by the cross peaks observed, at δ 1.16/16.99 and δ 1.22/16.99 in Figure 9a, for the rhamnose residue. These findings are highly consistent with the monosaccharide composition analysis results. The methyl proton signal of O-acetyl observed in the δ 1.90–2.20 region of the 1H-NMR spectrum, along with multiple signal peaks, indicated that the acetyl group was substituted at different positions of the sugar residues in the sugar chain. In the carbon spectrum, the absence of significant methyl signals within the acetyl group at δ 20.00 [38,39], coupled with the low-intensity peaks in the hydrogen and carbon spectra, suggested that the polysaccharide sample underwent a low degree of acetylation. The characteristic absorption peak observed at δ 53.24 represents the methyl carbon attached to the C-6 position in the methyl-esterified galacturonic acid residue. The cross peak of signals was detected on the spectrum at δ 3.72/53.24, with δ 3.72 being the methyl proton signal of methyl ester (−COOMe) (Figure 9a). Furthermore, the cross peak at δ 3.72/171.16 on the spectrum corresponded to the characteristic absorption peak at the C-6 position in the esterified galacturonic acid residue, near δ 171.16 on the carbon spectrum (Figure 9c). In contrast, the weaker characteristic signal near δ 175.31 indicated the presence of an unesterified galacturonic acid residue [40,41].

Based on the results of the monosaccharide composition and methylation analyses of the polysaccharide sample (Figure 8a,b and Figure 9a,b), the NMR spectra revealed several hetero-head signals. This data, in the light of the literature [36,40,42,43,44,45,46], suggested stronger signals at δ 98–102 in the hetero-head region, which presumably belonged to the hetero-head carbon signal of the sugar residue α-GalpA. A strong signal was observed near δ 102–105 in the hetero-head region, which presumably belonged to the hetero-head carbon signal of the sugar residue β-Galp. A signal was detected at δ 106–110 in the hetero-head region, which presumably belonged to the hetero-head carbon signal of the sugar residue α-Araf. The polysaccharide samples also contained several significant signal peaks in the multiple hetero-head region. These signals can be used for structural analyses. The chemical shifts of these hetero-head region signals were δ 4.87/100.76, δ 5.01/99.40, δ 5.09/98.75, δ 4.41/103.45, δ 4.53/103.85, δ 4.37/103.71, δ 5.15/109.63, δ 4.99/108.03, and δ 5.16/99.02. The attribution of the hetero-head signal was determined. Then, using the chemical shift data of similar sugar residue substitutions obtained through the 2D NMR spectra, combined with the 1D NMR spectra, and the results of the monosaccharide composition and methylation analyses against the relevant literature [36,37,38,39,40,41,42,43,44,45,46,47,48,49,50,51,52], the aforementioned sugar residues were labeled in the order as follows: MeGA1,4, GA1,4, GAt, X1,4, Gt, G1,3,6, At, A1,3, and Rha1,2,4(Rha1,2). The sugar residues corresponding to the reducing end group δ 4.53/96.45 were labeled as Rβ. Table 5 presents the findings on the attribution of the 1H and 13C chemical shift signals for the main types of sugar residues in the polysaccharide samples. According to the methylation analysis, the polysaccharide samples also contained 1,3,4-GalpA, 1,3-Galp, 1,6-Galp, 1,4-Glcp, T-Rhap, t-GlcpA, and other linkages, which displayed weak signals in regard to the NMR spectra, not attributable to 1H and 13C. The sequence of linkage between various sugar residues may be deduced based on the HMBC distant correlation and NOESY spectra. The HMBC distant correlation spectrum (Figure 9c) and NOESY spectrum (Figure 9d) of the polysaccharide samples revealed that the large molecular weight and low solubility of the polysaccharides presented with a few cross peak signals in the HMBC profile and coupling signals, which were mainly found in the NOESY profile. In the NOESY spectrum, (1) there is a cross (MeGA1,4 H-1/MeGA1,4 H-4) between H-1 (δ 4.87) and H-4 (δ 4.37) of the MeGA1,4, residue, which indicates the presence of a →4)-α-D-GalpA-6-OMe-(1→4)-α-D-GalpA-6-OMe-(1→ linkage. (2) There is a cross peak (GA1,4 H-1/MeGA1,4 H-4) between H-1 (δ 5.01) of the GA1,4 residue and H-4 (δ 4.37) of the MeGA1,4 residue, which indicates the presence of a →4)-α-D-GalpA-(1→4)-α-D-GalpA-6-OMe-(1→ linkage. (3) There is a correlation signal (Rha1,2,4 H-1/GA1,4 H-4) between H-1 (δ5.16) of the Rha1,2,4 residue and H-4 (δ 4.34) of the GA1,4 residue, which indicates the presence of a →2,4)-α-L-Rhap-(1→4)-α-D-GalpA-(1→ linkage, with the connecting point at O-4. (4) There is a correlation signal (GA1,4 H-1/Rha1,2,4 H-2) between H-1 (δ 4.93) of the GA1,4 residue and H-2 (δ 4.03) of the Rha1,2,4 residue, which indicates the presence of a →4)-α-D-GalpA-(1→2,4)-α-L-Rhap-(1→ linkage, with the connecting point at O-2. (5) There is a correlation signal (GA1,4 H-1/Rha1,2 H-2) between H-1 (δ 4.93) of the GA1,4 residue and H-2 (δ 4.03) of the Rha1,2,4 residue, which indicates the presence a →4)-α-D-GalpA-(1→2)-α-L-Rhap-(1→ linkage, with the connecting point at O-2. (6) There is a correlation signal (Gt H-1/Rha1,2,4 H-4) between H-1 (δ 4.53) of the Gt residue and H-4 (δ 3.64) of the Rha1,2,4 residue, which indicates the presence of a β-D-Galp-(1→ to →2,4)-α-L-Rhap-(1→ linkage, with the connecting point at O-4. (7) There is a correlation signal (G1,3,6 H-1/Rha1,2,4 H-4) between H-1 (δ 4.37) of the G1,3,6 residue and H-4 (δ 3.64) of the Rha1,2,4 residue, which indicates the presence of a →3,6)-β-Galp-(1→ linked to →2,4)-α-L-Rhap-(1→ linkage, with the connecting point at O-4. (8) There is a correlation signal (X1,4 H-1/Rha1,2,4 H-4) between H-1 (δ 4.41) of the X1,4 residue and H-4 (δ 3.64) of the Rha1,2,4 residue, which indicates the presence of a →4)-β-D-Xylp-(1→ linked to →2,4)-α-L-Rhap-(1→ linkage, with the connecting point at O-4.

**Figure 10:**
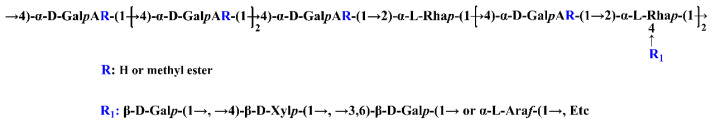
Predicted structure of the repeating units in AHP-3a.

The combination of the monosaccharide composition and methylation analyses and the polysaccharide 1D and 2D NMR information suggested that the study sample was a complex polysaccharide. This polysaccharide can largely be presumed to be composed of pectin with HG-type and RG-I-type structural domains containing side chains. The possible structural repeating units in these domains are illustrated in Figure 10.

### 2.9. Evaluation of In Vitro Antioxidant Activity

**Figure 11:**
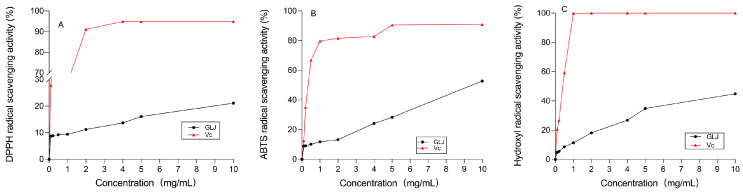
Antioxidant activity of AHP-3a. (**A**) AHP-3a at different concentrations DPPH radical scavenging activity assay. (**B**) AHP-3a at different concentrations ABTS radical scavenging activity assay. (**C**) AHP-3a at different concentrations Hydroxyl radical scavenging activity assay.

DPPH is commonly used to assess the free radical-scavenging activity of antioxidants; it is a stabilized free radical that can be reduced by accepting electrons or hydrogen in the presence of antioxidants. This is a common antioxidant method that indicates the hydrogen-donating capacity of a sample [53], which is often used to assess the free radical-scavenging activities of antioxidants [54]. Some of the hydrogen-donating polysaccharides can reduce stabilized DPPH radicals to yellow diphenylpyrazine [55]. The results on the DPPH radical-scavenging ability of AHP-3a are shown in Figure 11A. The ability of AHP-3a to scavenge DPPH radicals increased gradually at 0.1–10 mg/mL. The scavenging rate was found to be dependent on the concentration. At a dose of 10 mg/mL, the scavenging rate of DPPHS radicals was 21.10%, which was lower than that of Vc. It was found that polysaccharides exert antioxidant functions mainly because the amino acids in polysaccharides provide protons to electron-deficient free radicals. Previously, we employed the Sevag method to remove the protein from a crude polysaccharide and, then, used DEAE-52 and Sephadex G-100 columns for separation and purification, resulting in a low DPPH radical-scavenging rate for AHP-3a.

We performed ABTS radical scavenging to measure the total antioxidant capacity of natural products. To assess the total antioxidant capacity of AHP-3a, we considered the Vc group as the control group and then determined its ability to scavenge ABTS radicals. The results (Figure 11B) indicate a dose-dependent increase in scavenging in the concentration range of 0.1–10 mg/mL. This finding is consistent with the results of the DPPH radical-scavenging assay. Owing to its high GalA content, the ABTS radical-scavenging rate was 52.83%, which was higher than that of the DPPH radical-scavenging rate. Moreover, it was found that the active hydroxyl group of GalA in AHP-3a may play an important role in scavenging ABTS [50].

Hydroxyl radical scavenging is crucial for the body’s antioxidant mechanism, as otherwise, these highly aggressive radicals can induce an imbalance and cause damage to biomolecules [56]. AHP-3a had a pronounced dose-dependent impact on hydroxyl radical scavenging (Figure 11C). Remarkably, at 10 mg/mL, AHP-3a exhibited an outstanding 44.90% scavenging rate against hydroxyl radicals, which surpasses the scavenging rate against DPPH radicals at the same concentration.

The antioxidant activity assay showed that AHP-3a scavenges DPPH, ABTS, and hydroxyl radicals in a concentration-dependent manner. ABTS > hydroxyl radical > DPPH was the order of magnitude of the antioxidant activity of AHP-3a. It could scavenge ABTS and hydroxyl radicals more effectively, especially ABTS, which might be related to the honeycomb pores found in AHP-3a. In addition, GalA possesses antioxidant properties [57]. The analysis of the monosaccharide composition revealed that the largest monosaccharide fraction in AHP-3a was GalA (35.4%), suggesting that an increase in the antioxidant activity of AHP-3a may contribute to a higher GalA concentration. In the polysaccharide samples, factors such as the monosaccharide composition, Mw, and β-glycosidic bonds were also correlated with the antioxidant activity.

Owing to the complex antioxidant mechanisms of polysaccharides, further studies are warranted to correlate their structural features with their antioxidant capacity. Therefore, herbal polysaccharides are a potential source of antioxidants. AHP-3a possesses the ability to scavenge free radicals and possesses certain antioxidant activity. The antioxidant mechanism of AHP-3a was realized by directly acting on free radicals, such as ABTS, hydroxyl radicals, and 1,1-diphenyl-2-picrylhydrazyl (DPPH). This study revealed structural information on acidic polysaccharides in galangal, thereby providing new insights and suggesting that it may have the potential to be developed as a natural antioxidant.

### 2.10. Vitro Anticancer Activity

**Figure 12:**
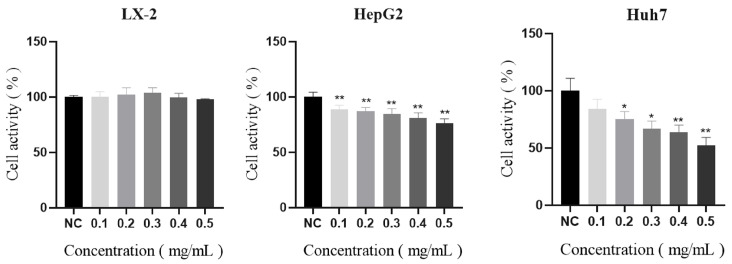
The effects of 0.1, 0.3, and 0.5 mg/mL AHP-3a on the viability of LX-2, HepG2, and Huh7 cells for 12 h. All values are expressed as the mean (x ± s) standard deviation (n = 3). * *p* < 0.05, ** *p* < 0.01, when compared with the CON group.

Polysaccharides may suppress the growth, migration, and invasion of cancer cells [58]. We assessed an AHP-3a-induced reduction in tumor cell proliferation by using three cell lines (i.e., LX-2, HepG2, and Huh7) to evaluate the anticancer efficacy of AHP-3a and confirm that it has anticancer potential. AHP-3a was not cytotoxic to normal cells (LX-2) at 0.1–0.5 mg/mL (Figure 12), albeit it did dose-dependently inhibit HepG2 and Huh7 cells. Huh7 cell viability was considerably suppressed at 0.5 mg/mL (*p* < 0.01), while HepG2 cell viability was significantly inhibited (*p* < 0.01). Subsequent studies used AHP-3a doses of 0.1, 0.3, and 0.5 mg/mL, because the capacity of HCC cells to proliferate was suppressed at these levels (*p* < 0.05).

**Figure 13:**
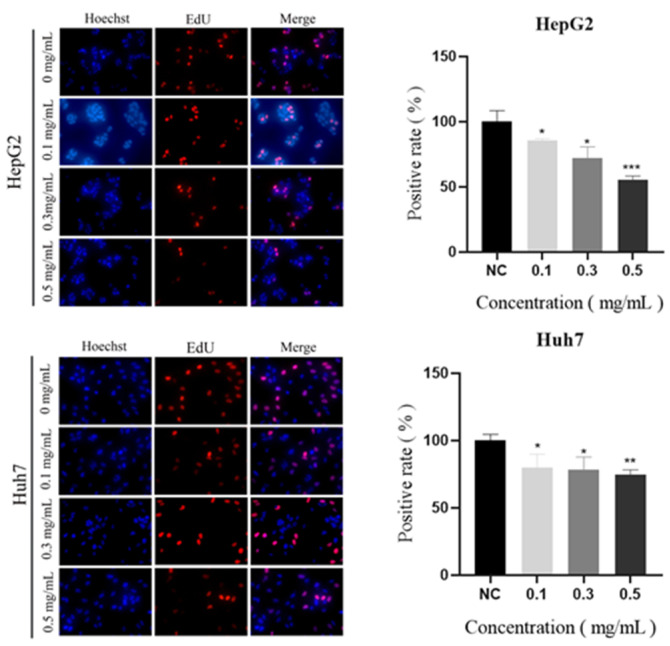
The effects of 0.1, 0.3, and 0.5 mg/mL AHP-3a on the cell proliferative capacity of Huh7 and HepG2; evaluation using the EdU assay. The experiments were independently repeated thrice, and the data are expressed as the mean ± SD. * Indicates the level of significance compared with the control group. * *p* < 0.05, ** *p* < 0.01, *** *p* < 0.001 when compared with the CON group.

The growth of cancer cells is uncontrollable by the body considering their endless capacity for reproduction. Therefore, preventing cancer cell multiplication is essential to combating cancer [20]. The degree to which the AHP-3a pure polysaccharide inhibited HCC cell proliferation was evaluated in this study. As shown in Figure 13, in the presence of AHP-3a, the EdU-positive staining of HepG2 and Huh7 cells decreased in a dose-dependent manner. When compared with the control group, 0.5 mg/mL of AHP-3a significantly inhibited Huh7 cell proliferation (*p* < 0.01) and significantly inhibited HepG2 cell proliferation (*p* < 0.001). Accordingly, the AHP-3a-purified polysaccharide exhibited an excellent inhibitory effect on the proliferation of HepG2 and Huh7 cells, signifying its good anticancer activity.

**Figure 14:**
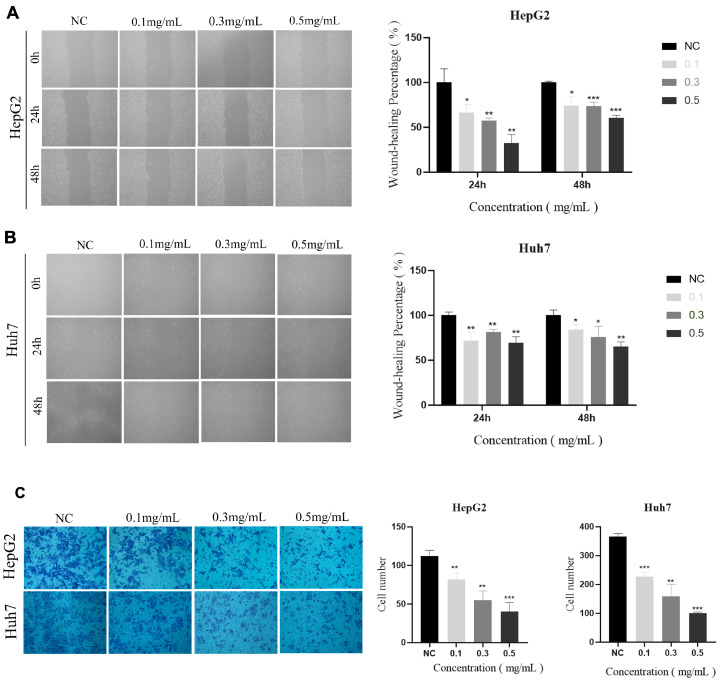
(**A**,**B**) The effects of 0.1, 0.3, and 0.5 mg/mL AHP-3a inhibit HepG2 liver cancer cell migration for 0 h, 24 h, and 48 h in wound healing. (**C**) The effects of 0.1, 0.3, and 0.5 mg/mL AHP-3a inhibit HepG2 liver cancer cell migration for 24 h in Transwell assays. All values are expressed as mean (x ± s) standard deviation (n = 3). * *p* < 0.05, ** *p* < 0.01, *** *p* < 0.001, when compared with the CON group.

Subsequently, the effects of 0.1, 0.3, and 0.5 mg/mL of AHP-3a polysaccharide on the invasive and migratory capacities of HepG2 and Huh-7 cells were investigated using wound healing and Transwell assays. The results of the wound-healing assay revealed that the cells treated with the AHP-3a polysaccharide had larger scratches than those in untreated HepG 2 cells and Huh-7 cells at 24 and 48 h (*p* < 0.05; Figure 14). Meanwhile, the Transwell assay revealed that the invasion rate of cells treated with the AHP-3a polysaccharide was significantly inhibited when compared with the corresponding control cells (*p* < 0.01). In addition, the number of invaded cells was significantly decreased due to treatment with 0.5 mg/mL of the AHP-3a polysaccharide (*p* < 0.001). These findings demonstrated that the AHP-3a polysaccharide significantly inhibited the invasion and migration of HepG2 and Huh-7 cells in a concentration-dependent manner. Notably, triple-helix polysaccharides have stronger anticancer activity [27], which may be a major contributing factor to the positive anticancer effects of the AHP-3a polysaccharide. Polysaccharides linked by β-(1→6)-glycosidic or β-(1→3)-glycosidic bonds possess significant anticancer action, and the glycosidic bonding mode has a key and significant role in antitumor activity [59,60]. The in vitro results, however, indicated that AHP-3a may greatly inhibit tumor cell growth and suppress proliferation/migration and the invasion of hepatocellular carcinoma cells with extraordinary anticancer potential; although the structural analyses did not reveal the presence of these two glycosidic bonds in large quantities. The current study only verified the effects of AHP-3a polysaccharides on hepatocellular carcinoma cell proliferation, migration, and invasion, and the mechanism by which AHP-3a regulates hepatocellular carcinoma in vivo warrants further investigation. Considering the complexity of the relationship between polysaccharide structure and function, future studies are necessary to understand this relationship. In conclusion, the AHP-3a polysaccharide is a potential anticancer component; although further studies are needed to understand the relationship between its structure and activity.

## 3. Materials and Methods

### 3.1. Materials and Chemicals

The galangal rhizome was cleaned and dried to a consistent weight, crushed, and then kept in a dry atmosphere. Salicylic acid was obtained from Tianjin Damao Chemical Reagent Co., Ltd. (Tianjin Damao Chemical Reagent Co., Ltd., Tianjin, China), and the following materials were used in this study: DEAE-52 cellulose (Greenherbs, Napier, New Zealand), Sephadex G-100 (Cytova, Marlborough, MA, USA), CCK8 assay kit (APExBIO, Houston, TX, USA), EdU cell proliferation kit (Beyotime, Nantong, China), hydroxyl free radical-scavenging capacity assay kit (Beijing Solarbio Science & Technology Co., Ltd., Beijing, China), and DPPH free radical-scavenging ability assay kit (Beijing Boxbio Science & Technology Co., Ltd., Beijing, China). Fetal bovine serum (FBS) and 1% penicillin–streptomycin were supplied by Guangzhou Saiguo Biotechnology Co., Ltd. (Guangzhou Saiguo Biotechnology Co., Ltd., Guangzhou, China). Transwell chambers and Matrigel matrix gel were purchased from Corning Life Sciences Co., Ltd. (Corning Life Sciences Co., Ltd., Jiangsu, China). Then, 4% tissue cell fixative solution and 0.1% crystal violet staining solution were purchased from Solarbio (Solarbio, Beijing, China). Unless otherwise stated, all chemicals used were of analytical grade.

### 3.2. Preparation, Isolation, and Purification of AHP

First, 1 kg of galangal rhizomes were crushed and extracted thrice, for 1 h each, in hot water (100 °C). All the extraction solutions were combined, and their pressure was reduced to a tenth of its initial volume. Then, 95% ethanol was added to 60% ethanol concentration at 4 °C and precipitated for 12 h. To obtain crude polysaccharides (AHP), the precipitated polysaccharides were dissolved in deionized water, deproteinized using the Sevag method, dialyzed, frozen, and dried.

The deproteinized AHP was dissolved in deionized water on a DEAE-52 cellulose column (2.6 × 30 cm) and gradually eluted with ultrapure water, 0.1 M NaCl, 0.2 M NaCl, and 0.3 M NaCl solution. An automatic partial collector (CBS-A Shanghai Jiapeng, Shanghai, China) was used to collect the effluent for 30 min per tube at a 0.5 mL/min flow rate. A polysaccharide content–tube number curve was plotted. The amount of sugar in each tube of eluent was calculated using the phenol–sulfuric acid method, and the polysaccharides were gathered according to the curve. Concentration, dialysis, and freeze drying were performed to achieve the preliminary purification of the galangal polysaccharide. The polysaccharide was further purified using a Sephadex G-100 column (2.6 × 30 cm), with ultrapure water serving as the eluent. The polysaccharide content–tube number curve was plotted after completing another round of sugar content measurement. After concentration and freeze drying, pure AHP was obtained.

### 3.3. Chemical and Monosaccharide Composition Analyses

The AHP-3a neutral polysaccharide content was determined using the phenol–sulfuric acid method [61]. The glyoxylate content was determined through the meta-hydroxyphenyl method, by using galactosyl glyoxylate as a standard [62]. Using myristic acid as a standard, the total phenolic content was determined through the indenol method [63]. The AHP-3a protein content was determined by using the Thomas Brilliant Blue method [64].

The AHP-3a monosaccharide composition was determined as per the method suggested by Ni et al. [21]. Briefly, AHP-3a (5 mg) was hydrolyzed using 3 mol/L TFA at 120 °C for 3 h. The hydrolyzed product was dried under a nitrogen-filled atmosphere, washed with methanol to remove residual TFA, and dissolved in water. Monosaccharide standards (such as fucose, rhamnose, arabinose, galactose, glucose, xylose, galacturonic acid, and glucuronic acid), as well as the polysaccharide samples, were measured using an ion chromatograph (ICS5000, Thermo Fisher, Norristown, PA, USA) with a Dionex Carbopac TMPA-20 analytical column (Thermo Scientific, Waltham, MA, USA) and an electrochemical detector. The mobile phases, consisting of A (H_2_O), B (15 mM NaOH), and C (15 mM NaOH with 100 mM NaOAc), were delivered at a 0.3 mL/min flow rate, at a column temperature of 30 °C.

### 3.4. Congo Red Analysis

The Congo red staining experiment was performed following the method by Nie et al. [65]. Briefly, 2.0 mL of 2.5 mg/mL AHP-3a solution and 2.0 mL of 40 μg/mL Congo red solution were mixed and different volumes of 1 mol/L NaOH solution were added at final concentrations of 0.5, 0.4, 0.3, 0.2, and 0.1 mol/L of NaOH. After incubating the mixture for 30 min at room temperature, the reaction mixture was run through a UV–vis spectrophotometer (JASCO V-650, JASCO, Easton, MD, USA) at 200–600 nm for full wavelength scanning and to record the maximum absorption wavelength (λmax).

### 3.5. UV–Vis Spectroscopy

A previously reported method was used, albeit with minor modifications [66]. Briefly, the dried AHP-3a was produced in deionized water at 1 mg/mL, both before and after purification. The product was then subjected to a full wavelength UV scan at 190–400 nm by using a UV spectrophotometer (JASCO V-650, JASCO, Easton, MD, USA).

### 3.6. Molecular Weight Determination

The High Performance Liquid Chromatograph (Waters 1525, UAS, Rydalmere, Australia) and a 2414 differential detector were used to assess the molecular weight distribution of AHP-3a through gel permeation chromatography (GPC) [67]. The polysaccharides were eluted using a PL aquagel -OH MIXED 8 μm (300 × 7.5 mm) column (Agilent, Santa Clara, CA, USA) at 30 °C, by using 0.2 M NaNO_3_ and 0.01 M NaH_2_PO_4_ (pH 7) as the mobile phases for 15 min, at a flow rate of 1 mL/min.

### 3.7. FT-IR Analysis

Previous research methods were referenced and fine tuned [68]. For the FT-IR spectroscopy analysis, 2 mg of the dried polysaccharide was thoroughly mixed with 100 mg of KBr and sliced into 2-mm-thick sections. The FT-IR spectra of the samples were then collected using an FT-IR spectrometer (Vector 33, Bruker, Billerica, MA, USA), in the range 4000–500 cm^−1^.

### 3.8. SEM Analysis

The AHP-3a surface morphology was characterized through scanning electron microscopy (SEM), by using a Carl Zeiss EVO 18 system. Before imaging, the samples were prepared through gold coating. The SEM images were collected at an accelerating voltage of 10.00 KV and different magnifications, such as 1000×, 100×, and 10×.

### 3.9. Methylation Analysis

The methylation analysis was performed according to the method by Taylor and Conrad (1972), albeit with some modifications [69]. Reduced AHP-3a was dissolved in DMSO containing NaOH. After 30 min of methylation with iodomethane, water, and dichloromethane were added to the mixture, mixed, and centrifuged. The aqueous phase was discarded through centrifugation. Washing was repeated thrice, and the liquid was evaporated to obtain the residue. The sample was hydrolyzed by adding 2 M TFA at 121 °C for 90 min and then evaporated. Then, 2 M ammonia and 1 M NaBD4 were added at room temperature to the hydrolyzed product, mixed, and reacted for 2.5 h. The reaction was terminated by adding acetic anhydride at 100 °C, and acetylation was performed for 2.5 h. The resulting product was added to dichloromethane, vortexed, mixed, and centrifuged, and the aqueous phase was discarded. The precipitate was washed with deionized water thrice. The dichloromethane layer was collected for gas chromatography–mass spectrometry analysis.

### 3.10. NMR Analysis

To investigate the chemical structure of AHP-3a, NMR analysis was performed according to the method described by Li et al. (2021) [33]. Briefly, 40–50 mg of the compound was accurately weighed in 600 μL of D_2_O. The solution was vigorously shaken, transferred into an NMR tube, and then analyzed using a 600 MHz Bruker NMR spectrometer (Bruker AVANCE HD III MHz Spectrometer, Bruker, Billerica, MA, USA), to acquire the 1H NMR, 13C NMR, HMBC, HSQC, COSY, and NOESY spectra.

### 3.11. Vitro Antioxidant Activity

To evaluate the antioxidant activity of AHP-3a, several methods were performed to assess the ability of AHP-3a to scavenge free radicals, including DPPH, ABTS, and hydroxyl radical-scavenging assays. AHP-3a was evaluated using ABTS and hydroxyl radical assay kits, wherein vitamin C (Vc) was used as the positive control. A modified version of the method by Tsai et al. [70] was employed for the DPPH assay. Briefly, AHP-3a solutions of varying concentrations (0.1–10 mg/mL) were prepared in deionized water and mixed with the DPPH–ethanol solution. The mixture was allowed to react in a dark environment at room temperature for 30 min. The absorbance values were measured at 517 nm, and the recorded values of the mixture were used. The DPPH radical scavenging (%) was determined as (−A_AHP-3a_ + A_control_)/A_0_ × 100; where A_0_, A_control_, and A_AHP-3a_ represent the absorbance values of the deionized water and the DPPH–ethanol mixture, various concentrations of the polysaccharide solution mixed with ethanol, and polysaccharide solution mixed with DPPH–ethanol, respectively.

### 3.12. Evaluation of In Vitro Anticancer Activity

#### 3.12.1. Cell Culture

Human normal hepatocyte LX-2 and hepatoma cells (HepG2, Huh7) were cultured in DMEM medium, containing 1% penicillin–streptomycin and 10% FBS, at 37 °C and under 5% CO_2_ in an incubator.

#### 3.12.2. Cell Viability Determination

Using the CCK8 approach, the activities of LX-2, HepG2, and Huh7 cells were assessed. The CCK8 cell counting kit was used to calculate their viability. Different concentrations (0, 0.1, 0.2, 0.3, 0.4, and 0.5 mg/mL) of AHP-3a were added to the cells (density: 10,000 cells/well) in a 96-well plate and incubated for 12 h. Then, 100 µL of a medium containing CCK8 was added to each well, and 100 µL of the original medium was discarded. The cells were incubated in the medium for an additional hour. The absorbance was measured at 450 nm by using the SpectraMax Plus Automatic Plate Reader (Molecular Devices, Sunnyvale, CA, USA). The experiment was repeated thrice.

#### 3.12.3. Cell Proliferation Measurement

Using the BeyoClickEdU-555 cell proliferation detection kit (ChemicalBook, Beijing, China), the cell proliferation ability was assessed. HCC cells were seeded into a 24-well plate, to which the test medication was added and incubated for 24 h. The cells were then fixed and dyed, according to the kit’s instructions. To visualize the proliferative ability of the cells, the cell images were captured using a fluorescence microscope (Zeiss, Oberkochen, Germany), after which, the proportion of Edu-positive cells (red) to all Hoechst-positive cells (blue) was calculated.

#### 3.12.4. Wound-Healing Assay

The cell migration capacity was analyzed, according to the method described by Wu et al. (2018) [71]. The bottom surface of the 6-well plate was uniformly scribed with a black marker, and the HepG2 and Huh7 cells were inoculated into the 6-well plate at the density of 6 × 10^4^ cells/well. When the cell fusion rate reached 90%, 3 lines were drawn vertically with the tip of the gun. The free cells were washed away with PBS, a serum-free medium containing different concentrations of galangal polysaccharides (0.1, 0.3, 0.5 mg/mL) was added, and pictures were taken under the microscope at 0, 24, and 48 h. The migration rate was calculated using the following formula: cell migration rate (wound healing rate) = (initial scratch area − scratch area at moment t)/initial scratch area × 100%.

#### 3.12.5. Transwell Assay

Referring to the research methodology by Wang et al. (2019) with slight modifications [72], the diluted Matrigel matrix gel was uniformly spread on the membrane surface of the upper chamber of the Transwell with a pre-cooled lance tip, and 50 μL of Matrigel matrix gel was added to each chamber and incubated in an incubator at 37 °C under 5% CO_2_ and saturated humidity for 2 h. HepG2 and Huh7 cells in the logarithmic growth phase were taken and pretreated with 0.1, 0.3, and 0.5 mg/mL of galangal polysaccharide for 24 h. The concentration of the treated HepG2 and Huh7 cells was adjusted to 1 × 10^6^ cells/mL. Then, 200 μL of serum-free cell suspension was added to the upper chamber of the Transwell. Then, 500 μL of DMEM medium containing 20% fetal bovine serum was added to each well in the lower chamber and incubated for 24 h. Then, the cells under the membranes were fixed with 4% tissue cell fixative for 30 min, rinsed with PBS twice, and then stained with 0.1% crystal violet staining solution for 15 min, followed by rinsing with PBS to remove the residual crystal violet staining solution in the chambers. PBS was used to rinse the residual crystal violet staining solution in the chambers. Finally, the uninvaded cells in the upper layer of the chambers were gently wiped off using a disposable cotton swab and placed in the shade to dry. Finally, the penetrating cells were photographed and counted in 3 randomly selected areas, under an inverted microscope.

### 3.13. Statistical Analysis

The acquired data were processed and analyzed using statistical software, such as GraphPad Prism 9.0 and Origin 2018. The results obtained were calculated and expressed as the mean ± standard deviation (SD). To assess the differences between the groups, *t*-tests were performed, with non-parametric tests being applied for variance. Multi-group comparisons, on the other hand, were performed using a one-way ANOVA. Moreover, *p* < 0.05 was considered to indicate statistical significance.

## 4. Conclusions

Natural products have always been a part of traditional Chinese medicine practice and they continue to play an important role in drug development. In this study, a natural acidic polysaccharide, AHP-3a, was purified by ethanol precipitation and extraction, using cellulose DEAE-52 and Sephadex G-100 columns. Based on the results of the monosaccharide analysis, infrared spectroscopy, and nuclear magnetic resonance (NMR) spectroscopy analysis, it was hypothesized that AHP-3a possessed the structural domains of HG and RG-1 and a high proportion of galacturonic acid. In vitro, AHP-3a demonstrated good DPPH, ABTS, and OH radical-scavenging activities, which significantly inhibited the proliferation of HepG2 and Huh-7 hepatocellular carcinoma cells, without inducing any toxicity to normal cells and significantly inhibited the invasion and migration of HepG2 and Huh-7 cells in a concentration-dependent manner. The results suggest that AHP-3a polysaccharide is a potential source of biologically active polysaccharides. In addition, the possible anticancer mechanism of AHP-3a deserves further investigation. In addition, it is necessary to establish relevant animal models to further explore the action mechanism of AHP-3a against hepatocellular carcinoma in vivo.

## Figures and Tables

**Table 1:** The chemical composition of AHP-3a isolated from *Alpinia officinarum*.

Neutral Sugar (%)	Uronic Sugar (%)	Protein (%)	Total Phenolics (%)
28.08 ± 0.13	45.93 ± 0.22	ND	8.16 ± 0.07

**Table 2:** Monosaccharide composition (molar ratio%).

Fuc	Rha	Ara	Gal	Glc	Xyl	Man	GalA	GlcA
0.5	8.9	16.9	21.4	3.1	11.8	0.0	35.4	2.0

**Table 3:** Molecular weight analysis of AHP-3a.

	Mn (Daltons)	Mw (Daltons)	MP (Daltons)	Polydispersity
AHP-3a	273,062	484,410	582,790	1.77

**Table 4:** GC–MS analysis of methylated AHP-3a.

Sugar Derivatives	Glycosidic Linkage	RT (min)	Molar Ratio (%)
1,4-di-O-acetyl-2,3,5-tri-O-methyl arabinitol	T-Araf	11.530	8.881
1,5-di-O-acetyl-6-deoxy-2,3,4-tri-O-methyl rhamnitol	T-Rhap	12.559	3.104
1,3,4-tri-O-acetyl-2,5-di-O-methyl arabinitol	1,3-Araf	14.344	1.264
1,2,5-tri-O-acetyl-6-deoxy-3,4-di-O-methyl rhamnitol	1,2-Rhap	15.321	5.061
1,4,5-tri-O-acetyl-2,3-di-O-methyl xylitol	1,4-Xylp	15.457	6.139
1,5-di-O-acetyl-2,3,4,6-tetra-O-methyl glucitol	T-GlcpA	16.576	6.390
1,5-di-O-acetyl-2,3,4,6-tetra-O-methyl galactitol	T-Galp	17.294	12.282
1,5-di-O-acetyl-2,3,4,6-tetra-O-methyl galactitol	T-GalpA	17.294	3.816
1,2,4,5-tetra-O-acetyl-6-deoxy-3-O-methyl rhamnitol	1,2,4-Rhap	18.200	9.370
1,4,5-tri-O-acetyl-2,3,6-tri-O-methyl galactitol	1,4-GalpA	19.597	32.634
1,4,5-tri-O-acetyl-2,3,6-tri-O-methyl galactitol	1,4-Galp	19.597	1.417
1,4,5-tri-O-acetyl-2,3,6-tri-O-methyl glucitol	1,4-Glcp	19.830	2.051
1,3,5-tri-O-acetyl-2,4,6-tri-O-methyl galactitol	1,3-Galp	20.108	1.835
1,5,6-tri-O-acetyl-2,3,4-tri-O-methyl galactitol	1,6-Galp	21.330	1.447
1,3,4,5-tetra-O-acetyl-2,6-di-O-methyl galactitol	1,3,4-GalpA	21.744	1.038
1,2,4,5-tetra-O-acetyl-3,6-di-O-methyl glucitol	1,2,4-GlcpA	22.340	0.786
1,3,5,6-tetra-O-acetyl-2,4-di-O-methyl galactitol	1,3,6-Galp	24.157	2.486

**Table 5:** Assignment of 1H and 13C NMR AHP-3a chemical shift values.

Glycosyl Residues		Chemical Displacement δ ()							
			1	2	3	4	5a/5b	6a/6b	-OMe
MeGA1,4	→4)-α-D-GalpA-6-OMe-(1→	H	4.87	3.63	3.92	4.37	5.06/5.00		3.72
		C	100.76	68.26	68.75	79.25	70.92	171.16	53.24
GA1,4	→4)-α-D-GalpA-(1→	H	5.01	3.63	3.92	4.34	4.67		
		C	99.94	68.26	68.75	77.76	71.47	175.31	
GAt	α-D-GalpA-(1→	H	5.09	3.75	3.81				
		C	98.75	68.75	69.05			175.31	
Rβ	→4)-β-D-GalpA	H	4.53						
		C	96.45					175.31	
X1,4	→4)-β-D-Xylp-(1→	H	4.41		3.61	3.66	3.30/4.03		
		C	103.45	73.34	75.49	76.16	63.14		
Gt	β-D-Galp-(1→	H	4.53	3.42	3.57	3.81	3.65	3.64/3.74	
		C	103.85	71.99	73.22	69.05	75.64	61.24	
G1,3,6	→3,6)-β-D-Galp-(1→	H	4.37	3.45	3.76				
		C	103.71	71.99	81.56				
At	α-L-Araf-(1→	H	5.15	4.12	3.85	4.04	3.57		
		C	109.63	81.82	77.09	84.24	62.87		
A1,3	→3)-α-L-Araf-(1→	H	4.99	4.03	3.99	4.06	3.57		
		C	108.03	81.67	84.24	81.41	62.87		
Rha1,2,4	→2,4)-α-L-Rhap-(1→	H	5.16	4.03	3.86	3.64		1.22	
		C	99.02	76.72	73.89	80.59		16.99	
Rha1,2	→2)-α-L-Rhap-(1→	H	5.16	4.03	3.86			1.16	
		C	99.02	76.72	73.89			16.99	

## Data Availability

Data are contained within the article.

## Abbreviations

GPC, gel permeation chromatography; IC, ion chromatography; SEM, scanning electron microscope; FT-IR, Fourier transform infrared spectroscopy; NMR, nuclear magnetic resonance; GalA, galacturonic acid; Gal, galactose; Ara, arabinose; Xyl, xylose; Rha, rhamnose; Glc, glucose; GlcA, glucuronic acid; Fuc, fucose; Man, mannose; HG, homogalacturonan; RG-I, rhamnogalacturonan-I; Vc, vitamin C; TFA, trifluoroacetic acid.
